# Regulation and Evasion of Host Immune Response by African Swine Fever Virus

**DOI:** 10.3389/fmicb.2021.698001

**Published:** 2021-09-08

**Authors:** Lei Wu, Bincai Yang, Xu Yuan, Jinxuan Hong, Min Peng, Ji-Long Chen, Zhongbao Song

**Affiliations:** Key Laboratory of Fujian-Taiwan Animal Pathogen Biology, College of Animal Sciences, Fujian Agriculture and Forestry University, Fuzhou, China

**Keywords:** African swine fever virus, regulation, immune response, interferon, inflammation, apoptosis

## Abstract

African swine fever (ASF) is an acute lethal hemorrhagic viral disease in domestic pigs and wild boars; is widely epidemic in Africa, Europe, Asia, and Latin America; and poses a huge threat to the pig industry worldwide. ASF is caused by the infection of the ASF virus (ASFV), a cytoplasmic double-stranded DNA virus belonging to the *Asfarviridae* family. Here, we review how the virus regulates the host immune response and its mechanisms at different levels, including interferon modulation, inflammation, apoptosis, antigen presentation, and cellular immunity.

## Introduction

Emerging and re-emerging viral pathogens present a huge threat to livestock and poultry breeding and human food safety. African swine fever (ASF) is an acute deadly hemorrhagic viral disease caused by highly contagious ASF virus (ASFV) in domestic pigs and wild boars and has a high mortality rate, approaching 100%. The disease often results in hemorrhagic necrosis of multiple organs, such as lymph nodes, kidneys, and heart, especially abnormal spleen enlargement and bleeding (Zhao et al., 2019; Sun et al., 2021). ASF was described in Kenya for the first time in 1921 and spread to Europe, South America, and Southeast Asia, causing enormous economic losses to the swine industry worldwide over the past century (Eustace Montgomery, 1921; Gaudreault et al., 2020). In China, virologically confirmed ASF was reported in Liaoning province for the first time recently in 2018 (Zhao et al., 2019).

ASFV belongs to the genus *Asfivirus* in the family *Asfarviridae* (Dixon et al., 2013). As a nucleocytoplasmic large DNA virus (NCLDV) (Iyer et al., 2001), ASFV has a fairly complex structure including a DNA-containing central nucleoid, a core shell, an inner lipid envelope, an icosahedral capsid, and an outer lipid envelope (Wang et al., 2019). The genome of ASFV is approximately 170∼194 kb and encodes 150∼170 proteins; however, the functionality of most of the proteins remains unclear (Cackett et al., 2020). Based on capsid protein p72 encoded by viral *B646L* gene, ASFV is divided into 24 different genotypes (Ge et al., 2018). In China, ASFV genotype II is highly prevalent and devastated the swine industry that resulted in a sharp increase in pork prices and has seriously affected people’s living standards (Chen et al., 2020). Therefore, there is an urgent need of developing effective vaccines and antiviral drugs. In the past decades, nonetheless, various vaccination strategies have been tried, including inactivated vaccines, DNA vaccines, subunit vaccines, and adenovirus-vectored vaccines; yet most of these vaccines failed to induce effective protective immunity against ASFV (Arias et al., 2017; Jancovich et al., 2018; Murgia et al., 2019; Sanchez et al., 2019; Sunwoo et al., 2019). However, live attenuated vaccines (LAVs), a type of vaccines prepared from pathogens that have been mutated and showed reduced toxicity but maintained antigenicity, have been demonstrated to induce protection against homologous challenge, but some problems still need to be addressed, including the emergence and spread of more virulent strains even after vaccination (Reis et al., 2016, 2017; Borca et al., 2020; Chen et al., 2020).

In this review, we summarize the recent research advances about ASFV and its proteins involved in the regulation of host immune responses at the molecular level (Table 1), and we discuss the implications of the underlying molecular mechanism of interaction between virus and host for the development of protective vaccines.

**TABLE 1:** Host immune responses known to be regulated by African swine fever virus (ASFV).

**Immune response**	**Viral genes**	**Immune elements and mechanisms**	**Impact on virulence**	**References**
Type I interferon response	A276R	Dampening type I IFN response by regulating IRF3	ND^*a*^	Correia et al., 2013
	A528R	Promoting the expression of ULK1 to degrade STING	Attenuated	Correia et al., 2013; Reis et al., 2016; Li et al., 2021
	MGF360-12L	Interacting with nuclear transport proteins importin α (KPNA2, KPNA3, and KPNA4) to disrupt NF-κB nuclear translocation	ND	Reis et al., 2016; Zhuo et al., 2020
	I329L	Inhibiting the crucial adaptor protein TRIF	ND	Correia et al., 2013; de Oliveira et al., 2011
	DP96R	Degradation of TBK1	Attenuated	Reis et al., 2017; Wang et al., 2018
Inflammatory response	L83L	Binding protein IL-1β	No reduction in virulence	Wang et al., 2018
	A238L	Inhibiting the activation of the NF-κB pathway	No reduction in virulence	Powell et al., 1996; Gomez et al., 1999; Salguero et al., 2008
	I226L, A151R, NP419L, QP383R	ND	ND	Song et al., 2020
Apoptosis	A179L	Bind to pro-apoptotic proteins (Bid, Bim, Bak, and Bax) to inhibit apoptosis	ND	Banjara et al., 2017; Galindo et al., 2008
	A224L	Activating NF-κB pathway to promote anti-apoptotic genes expression, e.g., IAP and Bcl-2 family proteins	No reduction in virulence	Nogal et al., 2001; Dixon et al., 2019; Neilan et al., 1997
	EP153R	Inhibiting the expression of caspase-3	ND	Granja et al., 2004; Hurtado et al., 2004
	E183L	Interacting with DLC8 to activate caspase-3 and caspase-9	ND	Hernaez et al., 2004
	DP71L	Recruit host phosphatase 1 (PP1) and remove the phosphorylation of eIF-2α to restore cellular protein synthesis to block CHOP activation suppressing apoptosis	No reduction in virulence	Zhang et al., 2010
	A238L	Inhibiting CaN to decrease apoptosis	No reduction in virulence	Wang et al., 1998
Antigen-presentation		Suppressing the expression of MHC-I/II, CD14, and CD16 and inhibiting the process of autophagy and apoptosis		Wang et al., 1998; Franzoni et al., 2017; Zhu et al., 2019; Lithgow et al., 2014
Cellular immune response (T and NK cells)		Decreased the numbers of CD8 + effector T cells, γδ T cells, and CD4 + T cells		Wardley and Wilkinson, 1980
		Enhancing the cytotoxicity of NK cells and the secretion of IFN-γ by non-virulent ASFV infected NK cells		Franzoni et al., 2017; Huhr et al., 2020; Leitao et al., 2001

*^*a*^ND means not done. ASFV regulates host immune responses, including the type I interferon response, the inflammatory response, apoptosis, antigen presentation, and cellular immune response. The related viral genes, targets and mechanisms, and the effect of deleting these viral genes on virulence are listed in the table.*

## African Swine Fever Virus Immune Evasion Mechanisms and Their Relationship to Live Attenuated Vaccines

### Inhibition of Type I Interferon Response and Signaling

The type I interferon (IFN)-mediated antiviral response plays an indispensable role in viral clearance. Host pattern recognition receptors (PRRs) can discern pathogen-associated molecular patterns (PAMPs), such as viral RNA and DNA (Rai et al., 2021). The cyclic GMP-AMP synthase (cGAS) is responsible for sensing viral DNA and then catalyzes the generation of cyclic GMP-AMP (cGAMP) in the presence of ATP and GTP. cGAMP binds to the stimulator of IFN gene-encoded protein (STING) to recruit TANK-binding kinase 1 (TBK1) to form a complex that activates various transcription factors, such as nuclear factor-kappa B (NF-κB) and IFN regulatory gene 3 (IRF3) (Xia et al., 2016; Margolis et al., 2017). After that, these phosphorylated transcription factors translocate into the nucleus to initiate the transcription of type I IFN and promote the expression of several IFN-stimulated genes (ISGs), which induce an antiviral state (Hopfner and Hornung, 2020).

Although the IFN-mediated immune response provides a formidable first line of defense, ASFV has evolved several strategies to antagonize the host IFN-mediated immune response to replicate and spread in the host population. Indeed, ASFV encodes numerous multifunctional proteins. These proteins can manipulate and evade the host antiviral response by highly specific interactions with the elements of the IFN signaling pathway. For instance, a previous study reported that attenuated NH/P68 ASFV could induce a higher level of type I IFN response than virulent 22653/14 ASFV, demonstrating that virulent ASFV could inhibit the expression of type I IFN genes (Razzuoli et al., 2020). In addition, the recombinant ASFV Pr4Δ35 strain with deletion of the six multigene family 360 (MGF360) and two MGF530-infected macrophages were identified as better IFN-α inducer. A microarray transcription analysis was carried out to compare the transcriptional profiles of Pr4Δ35 and parental ASFV Pr4. The result of microarray analysis showed that 38 genes were significantly upregulated at 3 h post-infection (hpi) and 6 hpi by Pr4Δ35 strain, and most of them were related to the type I IFN response, such as ISG15, ISG43, MxB, and IFITM3 (Afonso et al., 2004). Similarly, it was also reported that the deletion of five MGF360 and three MGF530 genes of virulent ASFV Benin 71/1 strain attenuated the virus, promoted the expression of IFN-β, and provided protective immunity in domestic pigs against Benin 71/1 infection (Reis et al., 2016). This study suggests that these genes are responsible for restraining the IFN-mediated antiviral response to contribute to efficient viral replication.

The A276R gene is a member of MGFs and, therefore, a potential IFN antagonist. To verify the IFN antagonism exerted by A276R, luciferase reporter assays of IFN-β, ISRE, and GAS reporter were performed. The results showed that A276R could inhibit the expression of IFN induced by poly I:C. It was revealed that A276R could inhibit IFN-β production by targeting IRF3, but not IRF7 and NF-κB (Correia et al., 2013); however, this study did not explain on the impact on virulence of A276R protein (Table 1). Other similar studies were also performed to evaluate the function of *MGF505-7R* (A528R) gene on the IFN signaling pathway. The results indicated that A528R can suppress the production of IFN-β; but unlike A276R, A528R protein inhibited IFN-β production by targeting mainly NF-κB. Moreover, co-transfection with ISRE or GAS reporters showed that the A528R protein distinctly downregulates the activities of both reporters stimulated by IFN-β and IFN-γ (Correia et al., 2013). However, the underlying precise mechanism of how these viral proteins inhibit IFN production remains largely unclear. A recent study showed that A528R can negatively regulate the cGAS-STING-mediated IFN signaling pathway by promoting the expression of autophagy-related protein ULK1 to degrade STING. Moreover, A528R-deleted ASFV had better IFN inducibility than wild-type ASFV and was fully attenuated in swine (Li et al., 2021). A previous study demonstrated that deletion of MGF360-12L led to upregulation of IFN-β and provided protective immunity against the challenge of virulent ASFV (Reis et al., 2016), suggesting that MGF360-12L could be a potential IFN antagonist. To verify this hypothesis, Zhuo et al. (2020) performed an in-depth study and found that MGF360-12L can suppress the transcription level and promoter activity of IFN-β and NF-κB. MGF360-12L could interact with nuclear transport proteins importin α (KPNA2, KPNA3, and KPNA4) at the nuclear localization signal domain to disrupt NF-κB nuclear translocation, resulting in the inhibition of type I IFN production. These results uncovered a new immune escape mechanism exerted by ASFV.

Toll-like receptor 3 (TLR3) is a PRR that could sense viral nucleic acid to activate IRF3 and NF-κB signaling pathway through a TRIF-dependent manner. Bioinformatics analysis indicated that I329L protein is a type I transmembrane protein containing four leucine-rich repeats (LRRs), which is a structural feature of TLR family members. In addition, three conserved sequences (box 1, box2, and box3) of the toll-interleukin-1 receptor (TIR) domain of TLR3 were also observed in the I329L protein. Thus, the author predicted that I329L may be a homolog of TLR3 and participates in regulating the TLR3-mediated signaling pathway (de Oliveira et al., 2011). Luciferase reporter assays showed that I329L inhibited the activation of the IFN-β promoter induced by TLR3 ligand poly I:C and ectopic expression of TRIF. Furthermore, the IFN-β production in the supernatant of I329L and TRIF co-transfected cells was evidently decreased (Correia et al., 2013). Considering that TRIF is also involved in the TLR4-mediated inflammatory response and IFN response, I329L would be predicted to regulate the activation of NF-κB. The results demonstrated that I329l can also inhibit the activation of NF-κB induced by LPS. These results showed that I329L may target TRIF to downregulate the host antiviral response (Correia et al., 2013).

In addition, Garcia-Belmonte et al. (2019) reported that attenuated NH/P68, but not virulent Armenia/07, could activate the cGAS-STING-IRF3 signaling pathway and induced a high level of IFN-β, which suggests that virulent Armenia/07 can regulate the cGAS-STING pathway. DP96R is a conserved early expression protein and an important virulence factor that has been predicted to be a potential protein involved in immune escape (O’Donnell et al., 2017). Wang et al. (2018) showed that DP96R can inhibit the IFN-β production induced by cGAS-STING. Transient transfection of DP96R suppressed the expression level of exogenous TBK1 and phosphorylated TBK1 in a dose-dependent manner. However, the study failed to explain the mechanistic basis of TBK1 degradation induced by DP96R and also did not determine the possible interaction between DP96R and TBK1.

### Regulation of the Inflammatory Response by African Swine Fever Virus

Inflammation is an important immune defense mechanism, which is beneficial for the host to resist the infection of pathogens. However, the imbalance of the inflammatory response can also lead to tissue and organ damage, promoting the occurrence of diseases. The NLRP3-mediated inflammatory response has been extensively studied. The NLRP3 inflammasome consists of NLRP3, ASC, Caspase-1, and NEK7, which can sense various types of exogenous and endogenous danger signals, such as viral nucleic acids, bacterial RNA, and endogenous damage-associated molecular patterns (DAMPs) (Zhou et al., 2011; Sharif et al., 2019). Activated NLRP3 recruits the adaptor protein ASC and interacts with the PYRIN domain, resulting in pro-caspase-1 cleavage into active form caspase-1. Then caspase-1 processes pro-IL-1β and pro-IL-18 to its biologically mature forms, IL-1β and IL-18, triggering inflammation-dependent cell death (Kelley et al., 2019).

Previous studies showed that virulent ASFV infection could promote the release of acute-phase proteins in serum, including serum amyloid A (SAA) and C-reactive protein (CRP), and increase the expression of a number of inflammatory cytokines, such as IL-1α, IL-1β, IL-6, and TNF-α (Sanchez-Cordon et al., 2007; Walczak et al., 2021). Similarly, Wang et al. (2020) found that virulent SY18 ASFV infection induced a robust and sustained elevation of pro-inflammatory cytokines (such as TNF-α, IL-1β, IL-6, IL-18, RANTES, and IP-10) from 3 days post-infection (dpi) and a secondary drastic increase including TNF-α, IL-1β, IL-6, and IL-8 and IL-10 at the terminal phase of infection in domestic pigs. This suggests that virulent ASFV infection can induce an excessive cytokine storm.

As a pleiotropic pro-inflammatory cytokine, IL-1β is mainly secreted by monocytes, macrophages, and lymphocytes and plays an important role in regulating host innate and adaptive immune responses. However, multifunctional proteins encoded by viruses can inhibit the inflammatory responses to create a better environment for virus replication. Borca et al. (2018) determined the binding between viral L83L protein and IL-1β by a yeast two-hybrid assay and speculated that L83L may have an important role in modulating the IL-1β pathway. Song et al. (2020) utilized a high-throughput screening system to identify viral proteins regulating the inflammatory response; and they identified I226L, A151R, NP419L, and QP383R proteins encoded by the virus as potential suppressors of the inflammatory response.

A238L is an analog of the inhibitory subunit of NF-κB α (IκB-α) that can inhibit the activation of the NF-κB pathway (Powell et al., 1996). Moreover, it was predicted that A238L may be involved in regulating the transcriptional activation of a multitude of NF-κB pathway-dependent genes. Among them, pro-inflammatory cytokines are critical molecules to recruit inflammatory cells to the infected sites, which help in eliminating pathogens. Studies have shown that in ASFV-infected macrophages, the transcription level of TNF-α was upregulated at 2–4 hpi but downregulated at later times (Gomez et al., 1999). Moreover, in phorbol myristate acetate (PMA)-activated macrophages, TNF-α and IL-8 levels were significantly suppressed at the early stages post-infection (Powell et al., 1996). Although macrophages and domestic pigs infected with an A238L-deleted ASFV strain increased the level of TNF-α, the replication and virulence of the virus were not affected (Salguero et al., 2008). This suggests that virus may encode other proteins, which could compensate for the loss of A238L.

### Modulation of Apoptosis by African Swine Fever Virus

Apoptosis-mediated cell death is a fundamental process of life that plays an important role in maintaining body homeostasis. However, it is also a host anti-viral defense strategy to eliminate infected cells. Unfortunately, many viruses have evolved numerous anti-apoptosis strategies to inhibit the activation of the apoptosis pathway by encoding corresponding proteins. Evidence indicated that at least five proteins encoded by ASFV have been involved in regulating apoptosis, e.g., A179L, A224L, EP153R, E183L, and DP71L.

The A179L protein belongs to the B-cell lymphoma-2 (Bcl-2) family and interacts with several pro-apoptotic proteins (Brun et al., 1996; Revilla et al., 1997; Banjara et al., 2017). The interaction between Bcl-2 family proteins leads to changes in mitochondrial outer membrane permeabilization (MOMP) and induces cell apoptosis. This process involves three types of proteins: (1) effectors (such as Bax and Bak) are a class of pro-apoptotic proteins. Activated effectors can form tunnels on the mitochondrial outer membrane, thereby promoting MOMP-mediated apoptosis (Bleicken et al., 2010). (2) Guardians (such as Bcl-2 and Bcl-xL) are a class of anti-apoptotic proteins that can bind effectors to inhibit apoptosis (Chipuk and Green, 2008). (3) Initiators (such as Bim and Bad) are divided into activators and sensitizers (Letai et al., 2002). The activator could promote the effector’s activity to induce apoptosis, and the sensitizer indirectly activates apoptosis by suppressing the function of the guardian. It is reported that A179L can bind to a broad range of pro-apoptotic proteins (Bid, Bim, Bak, and Bax) in a BH1 domain-dependent manner and inhibits cell apoptosis (Galindo et al., 2008; Banjara et al., 2017).

The ASFV A224L protein is an analog of the inhibitor of apoptosis (IAP) protein family; these family members contain a specific BIR motif and inhibit external stimulated apoptosis (such as by TNF-α) through interaction with caspase-3 (Nogal et al., 2001). In A224L-deleted ASFV strain-infected cells, the activity of caspase-3 was significantly increased and promoted the occurrence of cellular apoptosis (Nogal et al., 2001). Further studies indicated that overexpression of A224L protein could activate the NF-κB pathway, indicating that this viral protein may dampen apoptosis through activating some anti-apoptotic genes, such as IAP and Bcl-2 family proteins (Dixon et al., 2019). However, the deletion of *A224L* gene did not change the replication and virulence of ASFV in macrophages and domestic pigs (Neilan et al., 1997).

Virus infection can induce cellular stress, by activating protein kinase R-like endoplasmic reticulum kinase (PERK) and double-stranded RNA-dependent protein kinase (PKR), which in turn leads to phosphorylation of the translation initiation factor eIF-2α, reducing the synthesis of total protein in the cell. Subsequently, this upregulates expression of downstream transcription factors ATF4 and CHOP (CCAAT-enhancer-binding protein homologous protein), leads to an increase of pro-apoptotic proteins, and mediates cellular apoptosis (Harding et al., 2000; Young and Wek, 2016). The ASFV DP71L protein has a conserved C-terminal domain similar to HSV-1 ICP34.5 protein that can recruit host phosphatase 1 (PP1) and remove the phosphorylation of eIF-2α to restore cellular protein synthesis (Zhang et al., 2008). Then the activation of the transcription factor CHOP, which is normally induced by ER stress and mediates apoptosis, is blocked, leading to the suppression of cellular apoptosis (Rivera et al., 2007; Zhang et al., 2010). However, the deletion of *DP71L* gene (also named NL) from ASFV Malawi Lil20/1 isolate did not promote the phosphorylation level of eIF-2α and had no influence on virulence in domestic pigs (Zhang et al., 2010). This indicated that other viral proteins may compensate for the loss of DP71L (Dixon et al., 2004).

In addition, the C-type lectin EP153R-deleted ASFV infection can promote the expression of caspase-3 and cell death in several cell lines, and exogenous expression of EP153R could inhibit external stimulated or virus infection-induced apoptosis (Granja et al., 2004; Hurtado et al., 2004). A238L, an *analog* of IkB-α, was reported to bind to and suppress calcium/calmodulin-regulated phosphatase *calcineurin (CaN)* (Dixon et al., 2004). Moreover, CaN can dephosphorylate the anti-apoptotic Bcl-2 family protein BAD to promote apoptosis (Wang et al., 1998). Thus, it was speculated that A238L may have an anti-apoptotic function by inhibiting CaN. In contrast, viral *E183L* gene encoding structural protein p54 was shown to interact with the 8-kDa light chain cytoplasmic dynein (DLC8) to trigger activation of caspase-3 and caspase-9; and overexpression of p54 resulted in caspase-3 activation and apoptosis (Hernaez et al., 2004).

### Influencing the Function of Antigen-Presenting Cells

Monocytes, macrophages, and dendritic cells (DCs) are the principal antigen-presenting cells (APCs) that play an indispensable role in the innate and adaptive immune response for viral clearance, including ASFV. Therefore, a better understanding of the impact of ASFV infection on APCs is beneficial to develop a safe and effective vaccine against the virus (Gomez-Villamandos et al., 2013; Franzoni et al., 2018a). *In vitro* studies indicated that APC infection with diverse virulent strains of ASFV led to the differential expression of key cytokines and surface markers (Gil et al., 2008; Franzoni et al., 2017, 2018a). Zhu et al. (2019) found that ASFV Georgia 2007 strain infection could inhibit various immune and cellular processes, such as MHC class II-mediated antigen processing and presentation, M1 macrophage activation, and the processes of macrophage autophagy and apoptosis. However, Franzoni et al. (2018a) observed that attenuated ASFV isolate (BA71V and NH/P68) could suppress the expression of MHC class I on infected macrophages, while the virulent strains did not. ASFV infection could also downregulate the surface expression of CD14 and CD16 on macrophages (Lithgow et al., 2014; Franzoni et al., 2017; Zhu et al., 2019), which may result in the impairment of anti-microbial/viral activity and impact macrophage’s function. It suggests that ASFV has evolved mechanisms to subvert macrophages’ functions to escape host immune responses. Apart from this, ASFV infection can also modulate the secretion of cytokines in macrophages, including IFN-α, IFN-β, TNF-α, IL-1α, IL-1β, IL-12, IL-18, CCL4, CXCL8, and CXCL10 (Franzoni et al., 2018a; Zhu et al., 2019).

As professional APCs, DCs can detect, uptake, and process antigen and present the processed antigens to lymphocytes. In *in vivo* experiments, animals infected with highly virulent L60 isolate resulted in a significant reduction of the number of interdigitating DC in mandibular lymph nodes (Gregg et al., 1995). Recently, Franzoni et al. (2018b) investigated the interaction between porcine monocyte-derived DCs (MoDC) and different virulence strains of ASFV, including avirulent BA71V, low-virulent NH/P68, and the virulent 22653/14. They observed that all three isolates were able to replicate in immature MoDC, but BA71V and NH/P68 strains could hardly infect mature MoDC induced by IFN-α. Moreover, they found that only attenuated strains but not virulent strains could downregulate MHC class I expression on MoDCs, which was similar to Franzoni’s observation (Franzoni et al., 2017). They indicated that downregulation of MHC class I may result in NK cell activation *in vivo*, and NK cell activation was correlated to the protection against ASFV. In addition, none of three isolates activated cytokine responses (such as IFN-β, IL-1β, IL-6, IL-8, IL-18, and TNF-α) from both immature and matured MoDC induced by IFN-α/TNF-α.

### Regulating the Host Cellular Immune Response

A previous study indicated that ASFV infection can result in impaired responsiveness of cellular immunity, can enhance apoptosis of lymphocytes, and cause lymphopenia (Takamatsu et al., 2013). Wardley and Wilkinson (1980) firstly reported that ASFV-specific memory T cells could be induced by non-virulent virus infection and protected pigs against the homologous virus, but no ASFV specific lymphocyte proliferation was detected in the virulent virus-infected group (Wardley and Wilkinson, 1980).

T cells, such as cytotoxic T cells (CTLs), γδ T cells, and memory helper T cells, play a vital role in the cellular immune response to defend against ASFV infection. Highly virulent ASFV Armenia08 infection decreased the numbers of CD8 + effector T cells and all γδ T cell subpopulations, namely, CD2−CD8− T cells, CD2 + CD8− T cells, and CD2 + CD8 + T cells. Moreover, the level of perforin expression in T cells was significantly reduced in lymphoid organs at 5 dpi. Similarly, the total number of CD4 + T cells in the peripheral blood of domestic pigs was also decreased (Huhr et al., 2020).

NK cells exert a powerful antiviral effect during viral infection by directly killing virus-infected cells or by secreting cytokines (such as IFN and TNF-α) and chemokines. Evidence indicated that asymptomatic pig herds infected by NH/P68 strain, a naturally attenuated ASFV isolate that is non-hemadsorbing and of low virulence, significantly enhanced the cytotoxicity of NK cells and resisted the challenge of the virulent ASFV Lisbon 60 isolate (Leitao et al., 2001). However, the moderately virulent ASFV Malta 78 isolate exerted a marked suppressive effect on NK activity between 3 and 6 dpi (Norley and Wardley, 1983). In addition, NK cells infected with non-virulent ASFV OURT88/3 could also secrete high levels of IFN-γ (Takamatsu et al., 2013). These data suggest that NK cells can play an important role in protective immunity against ASFV.

### Live Attenuated Vaccines and Immunomodulation

Previous studies indicated that most of the vaccines, including inactivated vaccines, DNA vaccines, subunit vaccines, and adenovirus-vectored vaccines, are not effective. However, Goatley et al. (2020) recently showed eight ASFV genes that when delivered to domestic pigs using an adenovirus prime and modified vaccinia Ankara boost can protect pigs against a fatal disease caused by genotype I ASFV strain. Meanwhile, LAVs are currently demonstrated to be the most promising and best-positioned candidates for ASF control, including conventionally attenuated isolates and gene deletion mutants. Attenuated viruses generated by continuous passage *in vitro* have been successful to control numerous viral diseases (Minor, 2015). Krug et al. (2015) indicated that ASFV-G passaged 110 times in Vero cells has decreased replication ability in primary swine macrophage cultures but did not confer protection for immunized pigs against challenge with virulent parental ASFV-G. Full-length sequence analysis revealed the gradual accumulation of gene deletions in specific areas of the viral genome. However, a passage-attenuated ASFV Congo could provide effective immune protection against challenge with the virulent parental Congo strain, but the virulent Congo strain can persist for weeks in recovered pigs (Titov et al., 2017). Naturally, the non-virulent OURT88/3 strain belongs to the genotype I isolate. It was reported that OURT88/3 strain could confer protection for immunized pigs against challenge with virulent ASFV isolate, including genotype I Benin97/1 isolate and genotype X Uganda 1965 isolate, but it did not induce complete protection for all of the pigs and also had adverse effects (King et al., 2011). In addition, Sanchez-Cordon et al. (2017) showed that intranasal immunization with low and moderate doses of OURT88/3 could provide complete protection against challenge with virulent OURT88/1, and the protected pigs showed transient clinical symptoms and short moderate levels of the virus genome in blood samples, but the virus persisted for a long time in some animals. Similarly, the low virulence NH/P68 isolate was also confirmed to protect animals after challenge with homologous virulent genotype I L60 strain and heterologous virulent genotype II Arm07 strain (Gallardo et al., 2018).

Although these conventionally generated LAVs were effective to control the homologous virulent strains, they led to some undesired complications under the field conditions. Moreover, because of the difficulty in the identification of chronically infected pigs that might act as continuous reservoirs, naturally attenuated ASFV poses a great challenge to eradicate the virus in endemic areas. Hence, deleting special viral genes using the CRISPR–Cas9 system or recombination technologies might unlock new possibilities for effective vaccine development. Evidence demonstrated that IFN inhibitory genes are very important for the virus to escape the host immune response. Consequently, the deletion of these genes can attenuate the virus. Deleting some of these genes (such as MGF360 and MGF505 family proteins) could provide protective immunity for swine herds against the challenge of virulent ASFV (Reis et al., 2016; Sanchez-Cordon et al., 2018; Chen et al., 2020). CD2V is a glycoprotein that could inhibit lymphocyte proliferation *in vitro*. A previous study indicated that the deletion mutant virus BA71ΔCD2 can confer protection against the challenge of the virulent ASFV, including the parental BA71, the heterologous E75, and Georgia 2007/1 strain (Monteagudo et al., 2017). However, Gladue et al. (2020) found that deleting *CD2V* gene significantly decreased the protective potential of the vaccine candidate ASFV-G-Δ9GL. Aiming to diminish adverse reactions and develop safe and effective vaccines, Gallardo et al. (2018) deleted A238L, A224L, A276R, and EP153R from parental virus NH/P68 and found that all of the mutant viruses could be fully protective against homologous challenge with L60 but failed to protect against genotype II Arm07, which is in contrast to the parental virus NH/P68. O’Donnell et al. (2015, 2017) reported that low doses (10^2^–10^3^ HAD_50_) of ASFV-G-Δ9GL could only result in partial protection against challenge with parental viruses, but the double-gene deletion recombinant virus ASFV-G-Δ9GL/ΔUK can offer increased safety and protection against homologous challenge.

## Conclusion and Perspectives

ASFV is a devastating veterinary pathogen, and the ASF outbreaks in farms remain often out of control in most countries. Although ASF was described firstly in Kenya a century ago, the research on the pathogenesis and immunity to ASFV infection is still lagging behind; particularly, the immunosuppressive mechanism exerted by ASFV remains largely unexplored, which seriously affects vaccine development and the control of the disease. The typical feature of ASFV infection is to induce an impaired host immune response that leads to rapid virus replication and acute illness in domestic pigs.

Currently, based on the available data, the fundamental roadblock to generate safe and effective LAVs is to explore the unknown functions of viral genes and complex host–virus interactions of various ASFV genotypes. Thus, investigating the functions of viral genes and the interaction between host and ASFV and clarifying the mechanisms of host immune response regulated by ASFV can pave the way to develop safe and effective vaccines.

In recent years, omics technology has been widely used to investigate the interaction between host and pathogens, and this will help enhance our knowledge. Performing omics research on ASFV, including transcriptomics, proteomics, and modifiedomics, should reveal a multitude of new targets involved in virus entry, replication, budding, and immunosuppression, which would provide additional targets for the development of safe and effective gene-deleted vaccines and other strategies for virus control.

## Author Contributions

All authors reported in the manuscript have made substantial contributions to the work. Under the supervision of J-LC and ZS, LW and BY wrote this manuscript together. JH and MP were responsible for revising the manuscript. XY drew the table.

## Conflict of Interest

The authors declare that the research was conducted in the absence of any commercial or financial relationships that could be construed as a potential conflict of interest.

## Publisher’s Note

All claims expressed in this article are solely those of the authors and do not necessarily represent those of their affiliated organizations, or those of the publisher, the editors and the reviewers. Any product that may be evaluated in this article, or claim that may be made by its manufacturer, is not guaranteed or endorsed by the publisher.
